# Genetic association of IL17 and the importance of ABO blood group antigens in saliva to COVID-19

**DOI:** 10.1038/s41598-022-07856-3

**Published:** 2022-03-09

**Authors:** Nao Nishida, Masaya Sugiyama, Yosuke Kawai, Izumi Naka, Noriko Iwamoto, Tetsuya Suzuki, Michiyo Suzuki, Yusuke Miyazato, Satoshi Suzuki, Shinyu Izumi, Masayuki Hojo, Takayo Tsuchiura, Miyuki Ishikawa, Jun Ohashi, Norio Ohmagari, Katsushi Tokunaga, Masashi Mizokami

**Affiliations:** 1 Genome Medical Science Project, National Center for Global Health and Medicine, 1-7-1 Kohnodai, Chiba, Ichikawa 272-8516 Japan; 2 Genome Medical Science Project, National Center for Global Health and Medicine, Shinjuku-ku, Tokyo 162-8655 Japan; 3 Department of Biological Sciences, Graduate School of Science, The University of Tokyo, Bunkyo-ku, Tokyo 113-0033 Japan; 4 Disease Control and Prevention Center, National Center for Global Health and Medicine Hospital, Shinjuku-ku, Tokyo 162-8655 Japan; 5 Biobank, National Center for Global Health and Medicine, Shinjuku-ku, Tokyo 162-8655 Japan; 6 Department of Respiratory Medicine, National Center for Global Health and Medicine Hospital, Shinjuku-ku, Tokyo 162-8655 Japan

**Keywords:** Genetics, Risk factors

## Abstract

The outbreak of COVID-19 caused by infection with SARS-CoV-2 virus has become a worldwide pandemic, and the number of patients presenting with respiratory failure is rapidly increasing in Japan. An international meta-analysis has been conducted to identify genetic factors associated with the onset and severity of COVID-19, but these factors have yet to be fully clarified. Here, we carried out genomic analysis based on a genome-wide association study (GWAS) in Japanese COVID-19 patients to determine whether genetic factors reported to be associated with the onset or severity of COVID-19 in the international meta-GWAS are replicated in the Japanese population, and whether new genetic factors exist. Although no significant genome-wide association was detected in the Japanese GWAS, an integrated analysis with the international meta-GWAS identified for the first time the involvement of the *IL17A/IL17F* gene in the severity of COVID-19. Among nine genes reported in the international meta-GWAS as genes involved in the onset of COVID-19, the association of *FOXP4-AS1*, *ABO*, and *IFNAR2* genes was replicated in the Japanese population. Moreover, combined analysis of *ABO* and *FUT2* genotypes revealed that the presence of oral AB antigens was significantly associated with the onset of COVID-19. *FOXP4-AS1* and *IFNAR2* were also significantly associated in the integrated analysis of the Japanese GWAS and international meta-GWAS when compared with severe COVID-19 cases and the general population. This made it clear that these two genes were also involved in not only the onset but also the severity of COVID-19. In particular, *FOXP4-AS1* was not found to be associated with the severity of COVID-19 in the international meta-GWAS, but an integrated analysis with the Japanese GWAS revealed an association with severity. Individuals with the SNP risk allele found between *IL17A* and *IL17F* had significantly lower mRNA expression levels of IL17F, suggesting that activation of the innate immune response by IL17F may play an important role in the severity of SARS-CoV-2 infection.

## Introduction

The outbreak of COVID-19 caused by infection with SARS-CoV-2 virus has become a worldwide pandemic, with 207 million cases and 4.36 million deaths globally as of Aug 2021. Since the first case of COVID-19 was reported in Japan in February 2020, 1,162,926 people have been infected and 15,439 people have died (as of Aug 16, 2021). To identify genetic factors involved in the onset and severity of COVID-19, genome analysis based on genome-wide association study (GWAS) has been carried out, including cohort-based GWAS^1,2^, GWAS by genetic testing companies^3^, and GWAS using biobanks^4^. The COVID-19 Host Genetics Initiative (COVID-19hg) is a multicenter study designed for this purpose, with the release 5 (January 18, 2021) revealing the results of an international meta-GWAS in which 50 research institutes or groups from all over the world have participated^5,6^. The Japan Coronavirus Taskforce is also participating in the COVID-19hg from Japan, and the international meta-GWAS includes 572 Japanese COVID-19 patients (155 of whom were severe COVID-19 patients) and 1705 healthy individuals. The international meta-GWAS comparing COVID-19 patients with the general population showed significant associations for nine genes: *LZTFL1*, *CCHCR1*, *FOXP4-AS1*, *TMEMS*, *ABO*, *OAS* family, *KANSL1*, *DPP9*, and *IFNAR2*. Seven genes, *LZTFL1*, *CCHCR1*, *VSTM2A*, *OAS* family, *TAC4*, *DPP9*, and *IFNAR2*, were significantly associated with severe COVID-19 patients compared with the general population. In July 2021, the COVID-19hg conducted an international meta-GWAS to increase the number of COVID-19 patients to 49,562, and newly reported the association of *SLC6A20* with COVID-19 susceptibility and *TYK2* with COVID-19 severity^2^. However, no significant SNPs were found in the international meta-GWAS of severe versus mild COVID-19 patients. Interestingly, it was reported that there exists a core haplotype in the 3p21.31 gene cluster including *LZTFL1* that is associated with severe COVID-19, and that the core haplotype was generated in Neanderthals and inherited by *Homo sapiens*^7^.

East Asians including Japanese, Koreans, and Chinese tend to have fewer COVID-19 cases than people in other countries. According to data compiled by Johns Hopkins University, the rates of COVID-19 infection in Japan, South Korea, and China were reported as 0.917% (123rd among 186 countries), 0.440% (137th), and 0.007% (185th), respectively, and the rates of death were 0.012% (123rd among 186 countries), 0.004% (148th), and 0.0003% (181st), respectively (as of Aug 16, 2021). The presence of genetic factors associated with the onset or severity of COVID-19 has been suggested, and *HLA* alleles with significant associations have been reported in Japanese^8,9^, Chinese^10^, and Hong Kong Chinese^11^ by *HLA* association analysis. A whole genome sequencing analysis of COVID-19 patients from the Chinese population has been reported, but no significant genes have been detected, because the number of patients analyzed was not sufficient and only included 64 severe patients^12^.

Here, we performed a genomic analysis based on GWAS in Japanese COVID-19 patients to determine whether genetic factors reported to be associated with the onset or severity of COVID-19 in the international meta-GWAS are replicated in the Japanese population and whether new genetic factors exist.

## Results

### Clinical characteristics of the 503 Japanese COVID-19 patients

**Figure 1:**
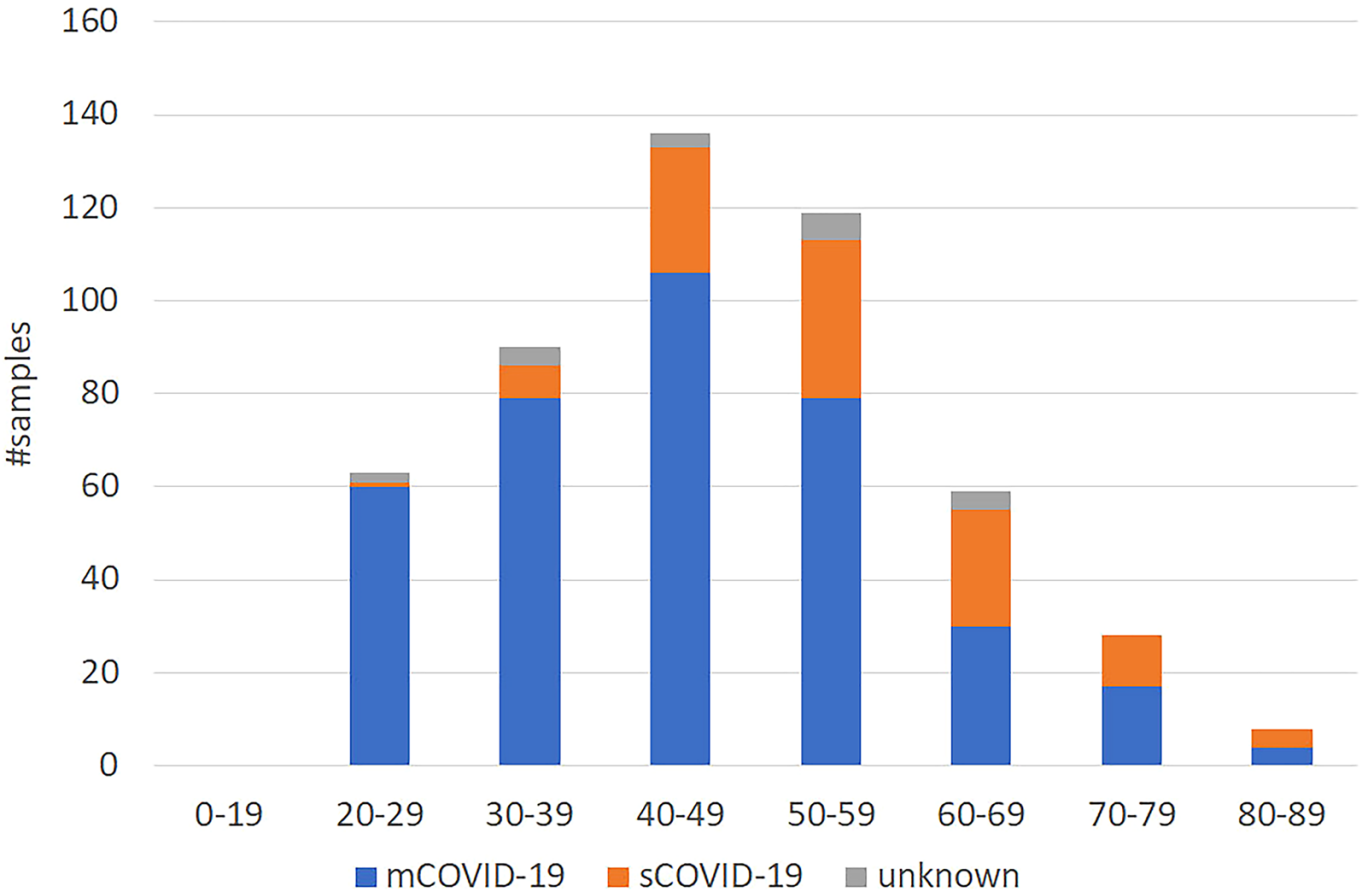
Age distribution of Japanese sCOVID-19 and mCOVID-19 patients.

Among the 503 Japanese COVID-19 patients in this study, 19 patients had recovered from COVID-19 but had no information on their severity. In addition to age and sex, the presence or absence of six underlying diseases (high blood pressure, dyslipidemia, type II diabetes (TIIDM), bronchial asthma, hyperuricemia, and obesity) in 484 patients with severity information is summarized in Table 1. Of the 109 severe COVID-19 (sCOVID-19) patients (see “Materials and methods” for clinical definitions), 86 were male and 23 were female, with an average age of 56.1 years, with the youngest at 27 years and the oldest at 88 years. Of the 375 mild COVID-19 (mCOVID-19) patients (see “Materials and methods” for clinical definitions), 178 were male and 197 were female, with an average age of 44.8 years, with the youngest at 20 years and the oldest at 89 years. Among the six underlying diseases, five were more common in patients with sCOVID-19 than in those with mCOVID-19, with hypertension accounting for 41.3% of sCOVID-19 patients (45 of 109) and 14.4% of mCOVID-19 patients (54 of 375), dyslipidemia for 23.9% (26 of 109) and 12.0% (45 of 375), TIIDM for 22% (24 of 109) and 5.6% (21 of 375), hyperuricemia for 19.3% (21 of 109) and 5.3% (20 of 375), and obesity for 15.6% (17 of 109) and 3.7% (14 of 375). For bronchial asthma, the opposite trend was observed: 3.7% (4 of 109) of sCOVID-19 patients and 6.1% (23 of 375) of mCOVID-19 patients. The age distribution of sCOVID-19 and mCOVID-19 patients is summarized in Fig. 1.

**Table 1:** Clinical background of all 503 COVID-19 patients.

Severity	#Total	Gender	Age		High blood pressure		Dyslipidemia		TIIDM		Bronchial asthma		Hyperuricemia		Obesity	
	n	M/F	Ave.	Min–max	n	%	n	%	n	%	n	%	n	%	n	%
sCOVID-19	109	86/23	56.1	27–88	45	41.3	26	23.9	24	22.0	4	3.7	21	19.3	17	15.6
mCOVID-19	375	178/197	44.8	20–89	54	14.4	45	12.0	21	5.6	23	6.1	20	5.3	14	3.7
Unknown	19	7/12	44.9	26–64	2	10.5	0	0.0	0	0.0	1	5.3	1	5.3	0	0.0

### GWAS using Japanese COVID-19 patients and healthy individuals

In a total of 462 Japanese COVID-19 patients who passed the sample quality control in GWAS, age and sex, the frequency of the six underlying diseases was compared between the two groups of patients with sCOVID-19 and those with mCOVID-19. Seven items, excluding bronchial asthma, were statistically significant in the univariate analyses by regression analysis on each of the 8 items (Table 2). In multivariate analysis by performing single regression with all 8 items, in addition to age and sex, hyperuricemia and obesity showed a statistically significant association with *P* < 0.05 (Table 2). These results suggest that elderly men with underlying diseases are at high risk of developing sCOVID-19.

**Table 2:** Association of eight clinical features in a comparison of patients with sCOVID-19 and mCOVID-19 in univariate and multivariate analysis.

	sCOVID-19 (n = 99)	mCOVID-19 (n = 347)	Univariate	Multivariate
Age, ave. (min–max)	56.0 (31–85)	45.1 (20–89)	**7.22E−12**	**6.95E−06**
Sex, M/F	78/21	166/181	**1.88E−08**	**6.25E−04**
High blood pressure, n (%)	42 (42.4%)	50 (14.4%)	**9.71E−09**	0.37
Dyslipidemia, n (%)	25 (25.3%)	43 (12.4%)	**2.78E−03**	0.59
TIIDM, n (%)	23 (23.2%)	20 (5.8%)	**2.01E−06**	0.15
Bronchial asthma, n (%)	4 (4.0%)	20 (5.8%)	0.49	0.79
Hyperuricemia, n (%)	21 (21.2%)	18 (5.2%)	**5.47E−06**	**1.60E−02**
Obesity, n (%)	15 (15.2%)	13 (3.7%)	**1.73E−04**	**2.10E−02**

*P* values that were statistically significant are indicated in bold.

**Figure 2:**
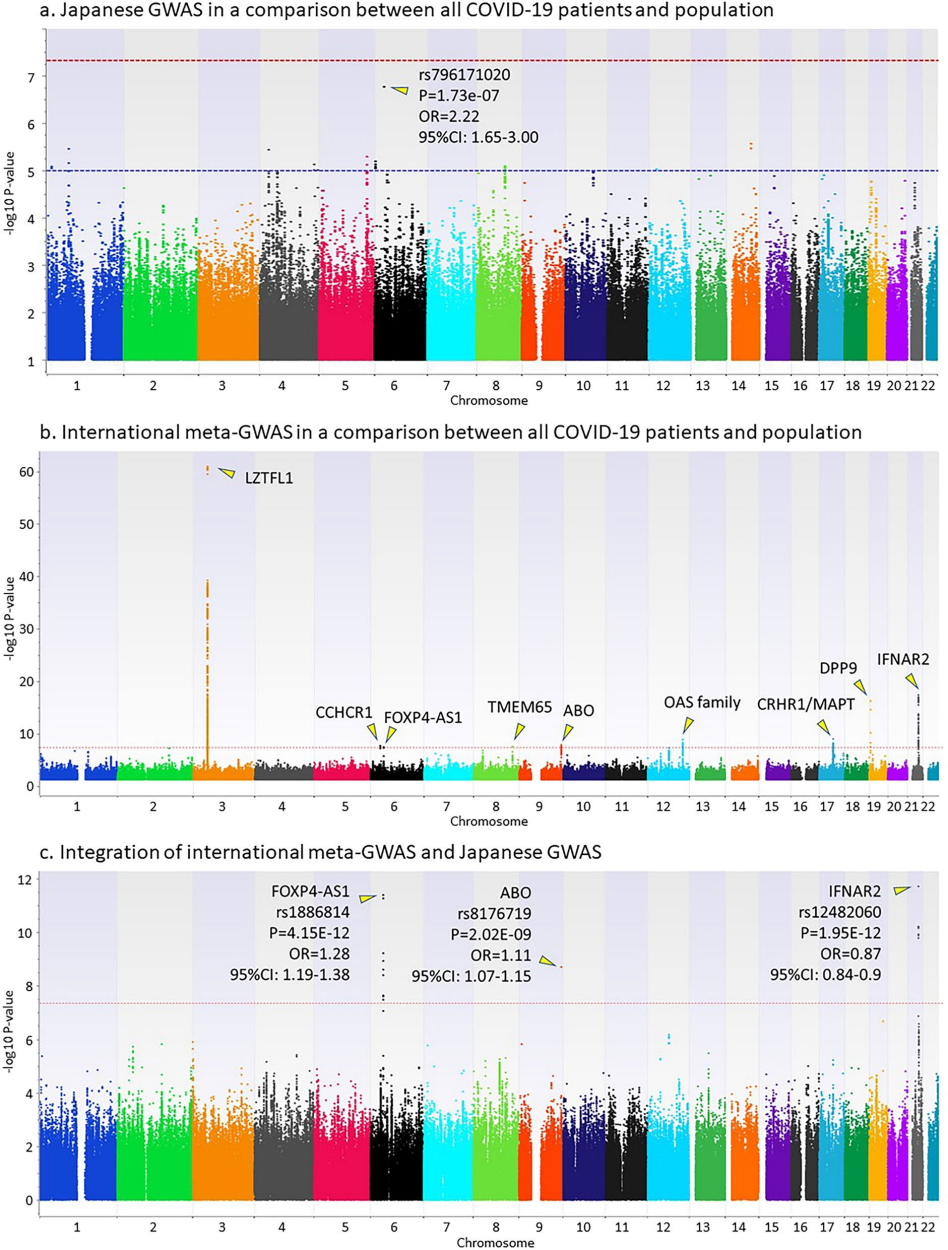
GWAS in a comparison between all COVID-19 patients and the general population. (**a**) Japanese GWAS comparing all COVID-19 patients with the general population. Regression analysis was applied using inferred sexes from genotypes on chromosomes X and Y. (**b**) International meta-GWAS comparing 7885 hospitalized COVID-19 patients with 961,804 general population members (mixed). The summary statistics were downloaded from the COVID-19hg. (**c**) Integration of international meta-GWAS and Japanese GWAS.

**Figure 3:**
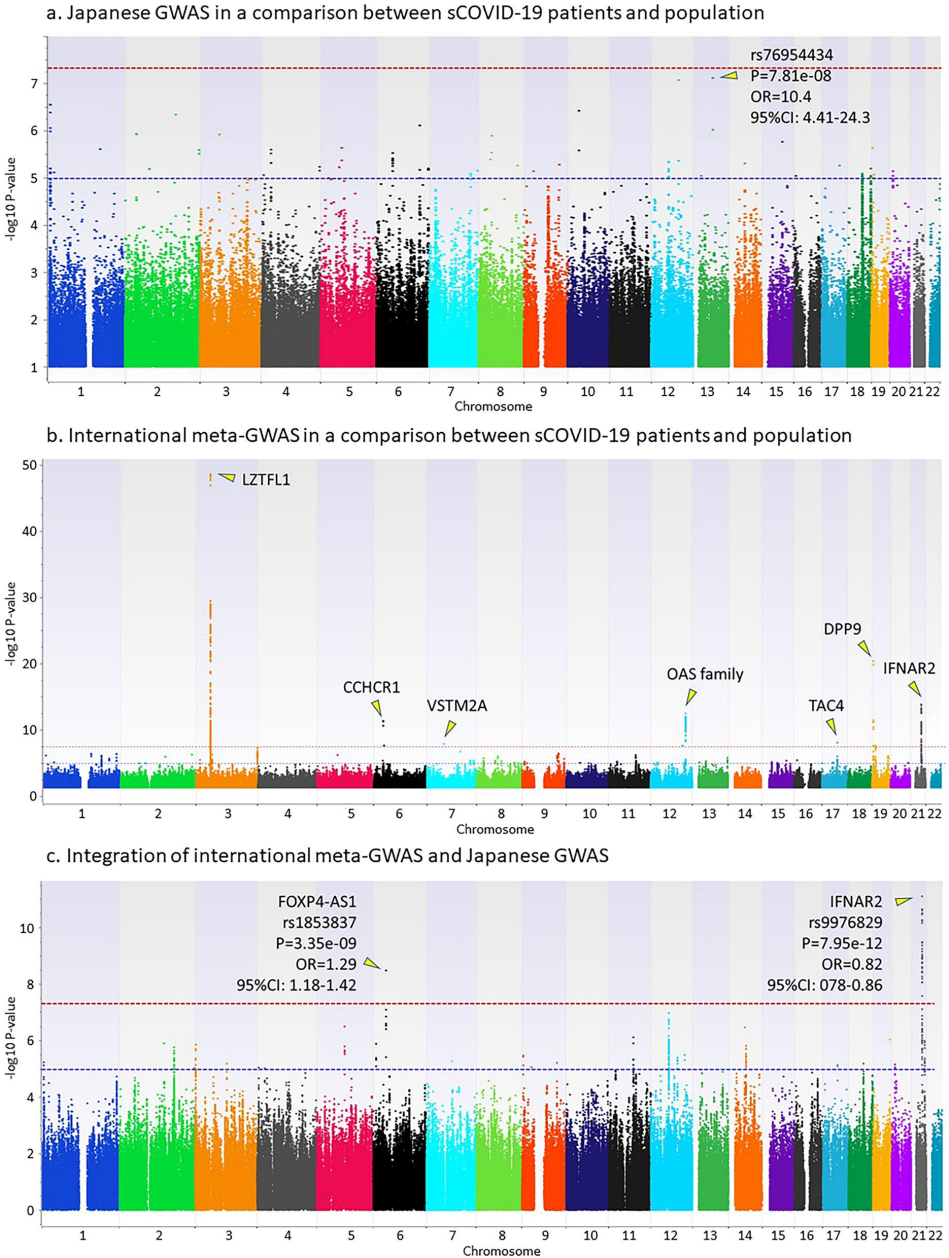
GWAS in a comparison between severe COVID-19 patients and the general population. (**a**) Japanese GWAS comparing sCOVID-19 patients with the general population. Regression analysis was applied using inferred sexes from genotypes on chromosomes X and Y. (**b**) International meta-GWAS comparing 4336 very severe respiratory confirmed COVID-19 patients with 623,902 general population members (mixed). The summary statistics were downloaded from the COVID-19hg. (**c**) Integration of international meta-GWAS and Japanese GWAS.

**Figure 4:**
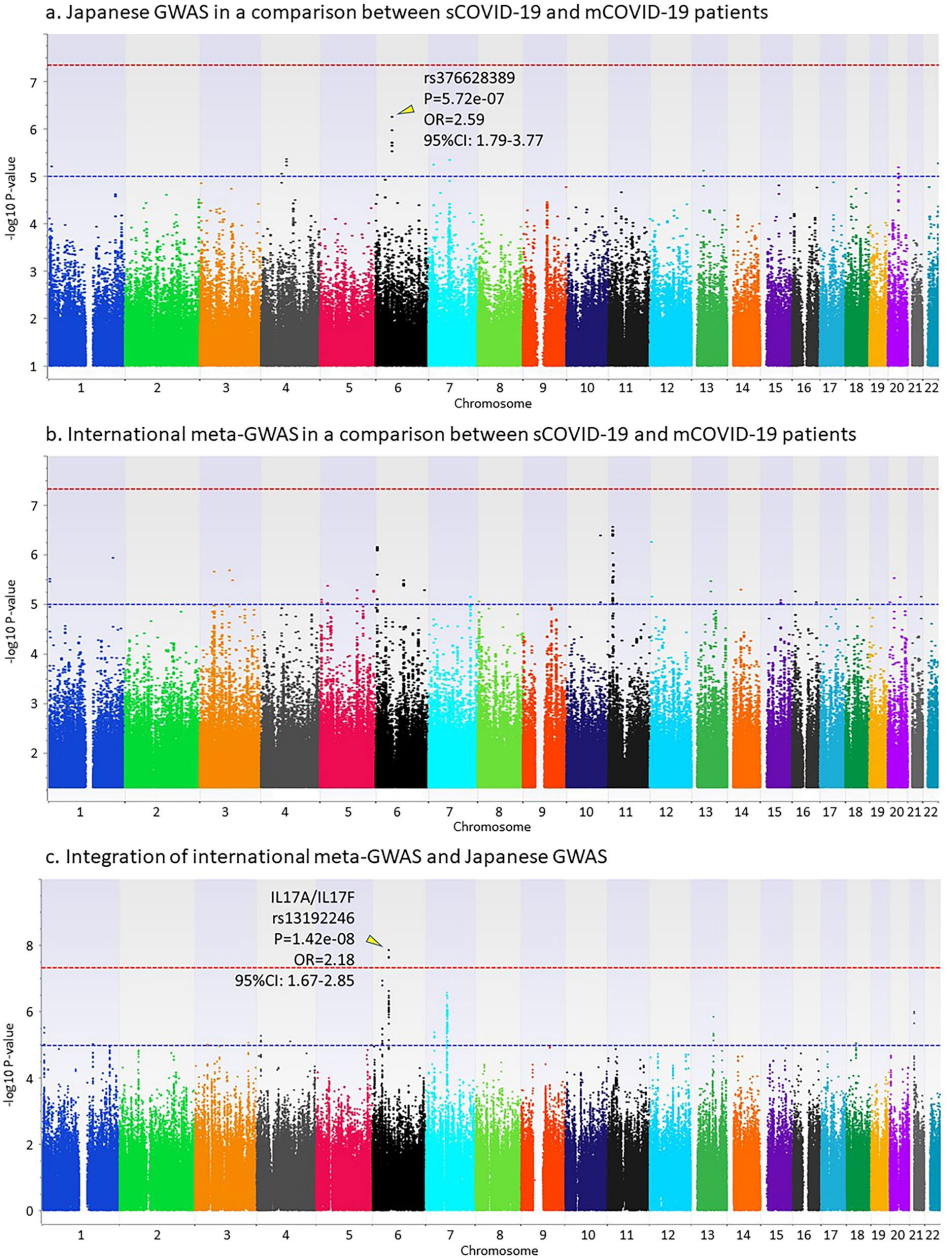
GWAS in a comparison between severe COVID-19 patients and mild COVID-19 patients. (**a**) Japanese GWAS comparing sCOVID-19 with mCOVID-19 patients. Regression analysis was applied using inferred sex, age, and presence or absence of six underlying diseases (high blood pressure, dyslipidemia, TIIDM, bronchial asthma, hyperuricemia, and obesity). (**b**) International meta-GWAS comparing 269 very severe respiratory confirmed COVID-19 patients with 688 non-hospitalized COVID-19 patients. The summary statistics were downloaded from the COVID-19hg. (**c**) Integration of international meta-GWAS and Japanese GWAS.

Genome-wide association studies using imputed genotypes from 462 Japanese COVID-19 patients and 1193 healthy individuals were carried out in three comparisons: (i) all COVID-19 patients and healthy individuals, (ii) sCOVID-19 patients and healthy individuals, and (iii) sCOVID-19 patients and mCOVID-19 patients. Regression analysis was applied using inferred sexes from genotypes on chromosomes X and Y for comparisons (i) and (ii), and using inferred sex, age, and presence or absence of the six underlying diseases for comparison (iii). Inflation factors in the three GWAS is 1.009, 1.007, and 1.031, respectively. Although no SNP reached the genome-wide significance level (*P* < 5e−8), the top hit SNPs were identified from (i) rs796171020 located on chromosome 6 (nearest gene is *DDX39BP2*) with a *P* value of 1.73e−07 and an OR of 2.22 (95% CI 1.65–3.00) (Fig. 2a); (ii) rs76954434 located on chromosome 13 (nearest gene is *LINC00355*) with *P* = 7.81e−08 and OR = 10.4 (95% CI 4.41–24.3) (Fig. 3a); and (iii) rs376628389 located on chromosome 6 (nearest gene is *IL17A*) with *P* = 5.72e−07 and OR = 2.59 (95% CI 1.79–3.77) (Fig. 4a).

### Integrated analysis to combine the Japanese GWAS and international meta-GWAS

Summary statistics of the international meta-GWAS were downloaded as the COVID-19 GWAS results (release date, Oct 20, 2020) at the time of analysis from the public download site supported by the NHLBI Intramural Research program and the NIH Biowulf high performance computing cluster (https://grasp.nhlbi.nih.gov/Covid19GWASResults.aspx). To conduct an integrated analysis of the Japanese GWAS comparing all COVID-19 patients with the general population, we downloaded international meta-GWAS statistics comparing 7885 hospitalized COVID-19 patients and 961,804 general population members from the COVID-19 host genetics initiative (COVID-19hg) (Fig. 2b). For an integrated analysis comparing sCOVID-19 patients and the general population, we downloaded international meta-GWAS statistics comparing 4336 very severe respiratory confirmed COVID-19 patients and 623,902 general population members from the COVID-19hg (Fig. 3b). For an integrated analysis comparing sCOVID-19 patients and mCOVID-19 patients, we also downloaded international meta-GWAS statistics comparing 269 very severe respiratory confirmed COVID-19 patients and 688 non-hospitalized COVID-19 patients from the COVID-19hg (Fig. 4b). Since the number of samples in the international meta-GWAS was overwhelmingly larger than that in the Japanese GWAS, the *P* value was integrated using Stouffer's Z-score (see “Materials and Methods” section for detail). Because the beta values and ORs of individual studies used in the international meta-GWAS are not publicly available, meta-analyses combined with data from individual studies have not been performed. Although the odds ratio (OR) cannot be accurately estimated because it is not a meta-analysis of individual studies, we examined whether the association of SNPs detected in the Japanese GWAS or international meta-GWAS was replicated, or whether a new SNP meeting the genome-wide significance level existed by combining the two.

Figure 2c shows the result of integrated analysis of the Japanese GWAS and international meta-GWAS comparing all COVID-19 patients and the general population. Three genetic regions, including *FOXP4-AS1*, *ABO*, and *IFNAR2*, showed significant associations in the integrated analysis. LocusZoom plots of 400 kb around the leading SNP for these three genetic regions are shown in Supplementary Figure 1a. Summary statistics showing significant associations in the integrated GWAS are summarized in Supplementary Table 1. These three genetic regions were originally detected in the international meta-GWAS, and it can be understood that the associations of these three genetic regions were replicated in the Japanese GWAS. *IFNAR2*, however, was not significant in Japanese GWAS because of the small number of samples.

Figure 3c shows the result of integrated analysis comparing sCOVID-19 patients and the general population. Significant associations from two genetic regions, including *FOXP4-AS1* and *IFNAR2*, were detected that were the same as those detected by comparison of all COVID-19 patients and the general population. LocusZoom plots of 400 kb around the leading SNPs for two genetic regions are shown in Supplementary Figure 1b. Summary statistics showing significant associations in the integrated GWAS are summarized in Supplementary Table 2. *FOXP4-AS1* was not significant in the original international meta-GWAS but met the genome-wide significance level by an integrated analysis with the Japanese GWAS. The SNP rs1853837, which was significant in the *FOXP4-AS1* gene, was not among the SNPs detected in the comparison of all COVID-19 patients and the general population, but the same tendency for the frequency of the risk allele to be higher in sCOVID-19 patients was observed. The *IFNAR2* gene was originally significant in the international meta-GWAS, and the association was replicated in the Japanese GWAS.

Figure 4c shows the result of the integrated analysis comparing sCOVID-19 and mCOVID-19 patients. Although no SNP satisfying the genome-wide significance level existed in either the international meta-GWAS or the Japanese GWAS, the integrated analysis showed that a SNP existing in the genetic region including *IL17A* and *IL17F* genes satisfied the genome-wide significance level (rs13192246, *P* = 1.42e−08). All four SNPs that met the genome-wide significance level in the integrated analysis showed a higher frequency of risk allele in the sCOVID-19 patients. LocusZoom plots of 400 kb around the leading SNP for the genetic region are shown in Supplementary Fig. 1c. Summary statistics showing significant associations in the integrated GWAS are summarized in Supplementary Table 3.

SNP functions including CADD score and RegulomeDB score for the associated SNPs in the integrated analysis are summarized in Supplementary Table 4.

### Association of combination of *ABO* and *FUT2* genotypes with the onset of COVID-19

The ABO blood type for 462 Japanese COVID-19 patients and 1193 healthy individuals was inferred from the results of genotype imputation. Haplotypes consisting of rs8176719, which determines the O blood type, and rs8176747, which determines the A and B blood type, were extracted from the results of genotype imputation. The genotypes of the ABO gene (AA, AO, BB, BO, AB, and OO) for each individual were then determined from these haplotypes. Chi-square tests were carried out in COVID-19 patients and health individuals using a 2 × 2 table with and without a specific blood type (i.e. A blood type, AA or AO). The association analysis of ABO genotypes with the onset of COVID-19 revealed that individuals with blood type A (i.e., AA and AO) were more likely to develop COVID-19 with a *P* value of 5.56e−03 and an OR of 1.36 (95% CI 1.09–1.70), while individuals with blood type O were less likely to develop COVID-19 with a *P* value of 2.73e−03 and an OR of 0.69 (95% CI 0.54–0.88) (Table 3). *FUT2* gene is essential for the synthesis of H antigen in saliva and determines whether it is secretor or non-secretor^13^. FUT2 secretor status is known to be determined by the genotype of rs1047781 for East Asian, i.e., A/A and A/T genotypes are blood type secretor and T/T is non secretor of blood type^14^. According to the rs1047781 genotype, the association between FUT2 secretor status and the onset of COVID-19 was examined, but no significant association was detected (Supplementary Table 5). To determine whether the presence or absence of A and B antigens in saliva is associated with the onset of COVID-19, the combined group of individuals with blood type O and non-secretor were compared with the other. The result showed that individuals with AB antigens in saliva were significantly more likely to develop COVID-19 with OR of 1.45 (95% CI 1.16–1.82), while those without AB antigens in saliva were significantly less likely to develop COVID-19 with OR of 0.69 (95% CI 0.55–0.86) (Table 4).

**Table 3:** Association of ABO blood type with development of COVID-19.

Blood type	COVID-19 patients		Healthy individuals		Chi *P*	OR	(95% CI)	
	(n = 461)		(n = 1193)					
	count	%	count	%			Lower	Upper
**AA, AO**	**199**	**43.2**	**427**	**35.8**	**5.56E−03**	**1.36**	**1.09**	**1.70**
BB, BO	101	21.9	270	22.6	7.52E−01	0.96	0.74	1.24
**OO**	**114**	**24.7**	**385**	**32.3**	**2.73E−03**	**0.69**	**0.54**	**0.88**
AB	47	10.2	111	9.3	5.80E−01	1.11	0.77	1.59

*P* values, statistically significant after correction of the significance level (*P* < 0.05/4), are indicated in bold.

**Table 4:** Association of a combination of ABO blood type and FUT2 secretor status with development of COVID-19.

ABO blood type + FUT2 secretor status	COVID-19 patients		Healthy individuals		*P*	OR	(95% CI)	
	(n = 461)		(n = 1193)					
	count	%	count	%			Lower	Upper
**OO + non-secretor**	**158**	**34.3**	**514**	**43.1**	**1.07E−03**	**0.69**	**0.55**	**0.86**
**Others**	**303**	**65.7**	**679**	**56.9**	**1.07E−03**	**1.45**	**1.16**	**1.82**

FUT2 non-secretor refers to a person who does not secrete ABH antigens in saliva. “Others” includes individuals with blood types A, B, and AB who have FUT2 secretor genotype (i.e. A/A and A/T). *P* values, statistically significant after correction of the significance level (*P* < 0.05/2), are indicated in bold.

## Discussion

The *IL17A*/*IL17F* gene region, which had the lowest *P* value in the GWAS comparing severe and mild COVID-19 patients in Japanese, met the genome-wide significance level as a result of an integrated analysis with the international meta-GWAS. Moreover, the association between *IL17A*/*IL17F* gene and COVID-19 severity was identified in a regression analysis that include age, sex, and underlying diseases as covariates, suggesting that *IL17A*/*IL17F* gene is involved in COVID-19 severity regardless of age, sex and underlying diseases. These results indicate that the association between COVID-19 severity and the *IL17A*/*IL17F* gene is not unique to the Japanese population and represents a novel disease-susceptibility gene detected by the integrated analysis with the international meta-GWAS. The eQTL data using the Genotype-Tissue Expression (GTEx) database for three SNPs that met the genome-wide significance level in the *IL17A*/*IL17F* gene region showed that IL17F mRNA expression levels were significantly reduced by carrying the risk allele associated with COVID-19 severity (Supplementary Figure 2). However, there is no registration of eQTL data for IL17A mRNA level in the GTEx database, and, the eQTL data for IL17F were for testis only. Although a direct comparison of IL17F mRNA expression between COVID-19 severe and mild patients will be required in the future, it is very interesting that the expression level of IL17F, which has been reported to be associated with protection against mucosal epithelial infection, may be significantly lower in severe COVID-19 patients^15,16^. IL17 has been reported to be produced from γδT cells and natural lymphocytes (innate lymphoid cells) in addition to the helper T cell subset Th17, and is involved in various diseases including multiple sclerosis, inflammatory bowel disease, and psoriasis^17^. There is an analysis using knockout mice of the functional difference between IL17A and IL17F^15^. It has been clarified that not IL17F but IL17A plays a main role in the development of autoimmune diseases such as arthritis and the allergic inflammatory response, and that IL17F is equivalent to IL17A or plays an important role in mucosal epithelium infection defense against *Staphylococcus aureus* and *Citrobacter rodentium*.

Next, we examined whether associated genes by the international meta-GWAS could be replicated in the Japanese GWAS. Of the nine genes found in the international meta-GWAS comparing all COVID-19 patients with the general population, three were replicated in the Japanese GWAS: *FOXP4-AS1*, *ABO*, and *IFNAR2*. The SNPs that exist in these three genetic regions met the genome-wide significance level in the integrated analysis, but none of the SNPs in the *IFNAR2* genetic region were significant (*P* > 0.05) in the Japanese GWAS. However, since the OR of each SNP was of the same direction as that in the international meta-GWAS, it is considered that sufficient detection power was not achieved in the Japanese GWAS due to the small number of cases. In the ABO genetic region, only one SNP (rs8176719) showed genome-wide significance in the integrated GWAS (Supplementary Figure 1), but this SNP is a well-known deletion that determines type O blood, indicating that even Japanese with type O blood are less likely to develop COVID-19. As a result of examining relationship between *ABO* genotype determined from GWAS data and the onset of COVID-19, individuals with blood type O were found to be less likely to develop COVID-19, and individuals with blood type A were more likely to develop COVID-19. Recently, it was reported that the SARS-CoV-2 virus can multiply in the mouth and spread through saliva^18^. And, the *FUT2* gene, which has been reported to be associated with norovirus infection^19^, is essential for the expression of ABH antigens in intestinal epithelial cells and saliva. Therefore, we focused on the *FUT2* gene, which determines the secretion type of ABH antigens in the oral cavity, and examined the association between individuals who express AB antigens in the oral cavity and those who do not. The results showed that individuals with oral AB antigens were more likely to develop COVID-19 and those without AB antigens were less likely to develop COVID-19. Although the *ABO* was found to be statistically significant in the integrated analysis of Japanese GWAS and international meta-analysis, these results suggest that the presence of oral AB antigens is actually important for COVID-19 onset. The associations of six genes including the *LZTFL1* gene, which showed the lowest *P* value in the international meta-GWAS, were not replicated in the Japanese GWAS. The SNPs with the lowest *P* value in each of these six genes in which the association was not replicated in the Japanese GWAS are summarized in Supplementary Table 6. Since SNPs genotyped in the international meta-GWAS were not included in the Japanese GWAS, it was not possible to compare the ORs directly, but there were no SNPs with *P* < 0.01. The SNP rs796171020 near the *DDX39BP2* gene, which had the lowest *P* value in the Japanese GWAS comparing all COVID-19 patients with the general population, did not show genome-wide significance even in the integrated analysis. These results indicate that three of the nine genes found in the international meta-GWAS also play important roles in the development of COVID-19 in Japanese.

*FOXP4-AS1* and *IFNAR2* genes also showed genome-wide significant associations in the integrated analysis comparing severe COVID-19 patients and the general population. Notably, *FOXP4-AS1* was not found to be associated with severe COVID-19 in the international meta-GWAS, but the integrated analysis with the Japanese GWAS revealed an association with severe COVID-19. Of the seven genes detected in the international meta-GWAS that compared severe COVID-19 patients and the general population, the SNPs with the lowest *P* values in six genes whose association was not replicated in the Japanese GWAS are summarized in Supplementary Table 7. As in Supplementary Table 6, it was not possible to compare ORs directly between SNPs from the international meta-GWAS and ones from the Japanese GWAS, but there were no SNPs with *P* < 0.01. These results suggest that three genetic factors are involved in the development of severe COVID-19 in Japan, including the *IL17A/F* gene, which was newly identified in this study.

This study revealed that the newly identified *IL17A/F* gene, which is associated with the severity of COVID-19, is a common genetic factor between populations. Because all the studied samples were collected from Japanese COVID-19 patients at discharge, it was not possible to determine whether IL17F could be used as a severity marker. If the measurement of serum IL17F levels confirms a decrease in IL17F levels in severe COVID-19 patients, it is expected to be an effective and important serum marker for predicting the severity of COVID-19. It is desirable to examine the effectiveness of IL17F as a diagnostic marker by collecting samples from COVID-19 patients upon admission in future.

## Materials and methods

### COVID-19 patients and clinical data

A total of 503 adult COVID-19 patients who were hospitalized as reported in the National Center for Global Health and Medicine (NCGM), or who were treated at home or in an accommodation facility, from January 30, 2020 to January 11, 2021 were recruited in this study. All COVID-19 patients and healthy individuals analyzed in this study were collected at NCGM separately from the Japan Coronavirus Taskforce. The presence or absence of underlying diseases in COVID-19 patients before their COVID-19 infection was obtained from questionnaire-based clinical information. All COVID-19 patients have not received a COVID-19 vaccine. In this study, COVID-19 patients were divided into two groups according to the latest (December 25, 2020) clinical guidelines from the Ministry of Health, Labor, and Welfare, Japan: sCOVID-19 and mCOVID-19. Patients who were defined as having sCOVID-19 showed clinical signs of pneumonia (fever, cough, dyspnea, and fast breathing) accompanied with one of the following symptoms: peripheral oxygen saturation (SpO2) ≤ 93% at room temperature, need for oxygen administration or a ventilator, and need of support from an extracorporeal membrane oxygenation (ECMO) device. Patients who were defined as having mCOVID-19 had any of the various signs and symptoms of COVID-19 (e.g. fever, cough, sore throat, malaise, headache, muscle pain, nausea, vomiting, diarrhea, loss of taste and smell), but did not have shortness of breath, dyspnea, or abnormal chest imaging. These criteria are in close agreement with the international standards set by the NIH (https://www.covid19treatmentguidelines.nih.gov/). To investigate the association between age, sex, and the presence or absence of underlying diseases and the severity of COVID-19 infection, a logistic regression analysis was conducted using individual variables as independent variables for a univariate analysis, and a logistic regression analysis was conducted using all eight variables as independent variables for a multivariate analysis.

This study was approved by the Ethics Committee of the National Center for Global Health and Medicine, and written informed consent was obtained from all COVID-19 patients (NCGM-G-003472). All methods were carried out in accordance with relevant guidelines and regulations.

### Japanese healthy individuals

Of the total of 1273 healthy adult Japanese individuals, 419 individuals (Tokyo Healthy Control, THC) residing in the Tokyo area before the COVID-19 outbreak were collected for widespread use in genome analysis. Informed consent was obtained from all 419 individuals. The remaining 854 individuals were purchased as Pharma SNP Consortium (PSC) samples from the Japan Health Sciences Foundation (JHSF). Human immortalized B cell lines were constructed from blood samples from about 1000 Japanese volunteers and were deposited in the Japanese Collection of Research Bioresources (JCRB)/JHSF, Health Science Research Resources Bank (HSRRB). The healthy individuals did not have information such as age or sex, so the inferred gender obtained from the GWAS data was used for statistical analysis.

### Genome-wide SNP genotyping and filtering samples

All 1776 genomic DNA samples from 503 Japanese COVID-19 patients and 1273 Japanese healthy individuals were genotyped using the Illumina Infinium Japanese Screening Array (JSA), according to the manufacturer’s instructions. Out of 1776 samples, 1759 had an overall call rate of more than 97%, and passed a heterozygosity check. Twenty-nine samples including 9 COVID-19 patients and 20 PSC healthy individuals were defined as related individuals (PI ≥ 0.1) in identity-by-descent testing and excluded from further analysis. A total of 75 samples, including 32 COVID-19 patients, 19 THC individuals, and 24 PSC individuals, were detected as outliers (IQR = 1.5) in multidimensional outlier detection and excluded from further analysis. Finally, 1655 samples including 462 COVID-19 patients and 1193 healthy individuals (consisting of 400 THC and 793 PSC) formed the same cluster using the first and second components in principal component analysis (Supplementary Figure 3) and were used for statistical analysis. Principal component analysis of these 1655 samples together with the data from the 1000 Genomes Project showed that two clusters of Japanese data (JPT) and our data were confirmed to be overlapped (Supplementary Figure 4). The average overall call rate for 1655 samples was 99.49% (minimum, 97.25; maximum, 99.73%). Genotypes of 342,721 SNPs selected by SNP filtering at a minor allele frequency of ≥ 5%, call rate of ≥ 95%, and HWE p value of ≥ 0.001 for controls were used for subsequent SNP genotyping imputation. Quality controls of samples and SNPs were carried out using the SNP & Variation Suite (SVS) software (Golden Helix, MT, USA) and PLINK 1.90 software (www.cog-genomics.org/plink/1.9/). Clinical background of 462 COVID-19 patients who passed the sample quality control in GWAS was summarized in Supplementary Table 8.

### SNP genotype imputation and statistical analysis

Genotype imputation was performed on the filtered SNP array data using BEAGLE 5.1^20^. Genotype data in VCF format were processed to match the reference panel using the conform-gt program, and then genotype imputation was performed using BEAGLE 5.1 with default settings. The reference panel for genotype imputation was made in-house. This panel comprises 9338 haplotypes from 4669 individuals from diverse populations including 2493 individuals from the International 1000 Genomes^21^, 820 individuals from the Human Genome Diversity Project^22^, 278 individuals from the Simons Genome Diversity Project^23^, 90 samples from the Korean Personal Genome Diversity Project^24^, and 1026 Japanese individuals from Biobank Japan. The Biobank Japan data are approved controlled access data from NBDC human data (JGAS000114), and the others were downloaded from public databases. Imputed variants (SNPs and indels) with low quality (DR2 < 0.5) were filtered out, and genotypes were hard called with the highest genotype probability, and genotype probabilities of less than 0.9 were considered no calls. The in-house reference panel for genotype imputation contains a total of 67,875,711 SNPs, a total of 15,167,811 SNPs were left after imputation, and 52,707,900 SNPs were excluded as low quality. In our previous report, we confirmed that the accuracy of the imputation does not change by using a reference panel composed of various populations when performing imputation on GWAS data only for Japanese^25^.

Statistical associations of COVID-19 with variants were tested using logistic regression. Variants with a minor allele frequency less than 1% or low call rate (< 95%) or HWE p-value < 1e−06 were excluded from the analysis. An additive genetic affect was assumed. Genome-wide association tests were conducted with plink 1.9. The genome-wide significance level in the Japanese GWAS was *P* < 5e−08, and the significance level for replication in the Japanese GWAS of SNPs that showed genome-wide significance in the international meta-GWAS was *P* < 0.05.

### Integrated statistical analysis to combine the Japanese GWAS with the international meta-GWAS

To combine the *P* value of Japanese GWAS with that of the international meta-GWAS, Stouffer's *Z*-score method was used in this study. In brief, the one-sided right-tailed *P* value was calculated from a two-sided *P* value in the international meta-GWAS based on the direction of association detected in the Japanese GWAS. Next, using the standard normal cumulative distribution function, the *Z*-score was calculated from the one-sided right-tailed *P* value. Then, *Z*-scores of the Japanese GWAS and of the international meta-GWAS were combined using the following equation:

$${{\varvec{Z}}}_{\mathbf{c}\mathbf{o}\mathbf{m}\mathbf{b}\mathbf{i}\mathbf{n}\mathbf{e}\mathbf{d}}=\frac{{{\varvec{Z}}}_{\mathbf{J}\mathbf{a}\mathbf{p}\mathbf{a}\mathbf{n}\mathbf{e}\mathbf{s}\mathbf{e}}+{{\varvec{Z}}}_{\mathbf{i}\mathbf{n}\mathbf{t}\mathbf{e}\mathbf{r}\mathbf{n}\mathbf{a}\mathbf{t}\mathbf{i}\mathbf{o}\mathbf{n}\mathbf{a}\mathbf{l}}}{\sqrt{2}}$$

The two-sided *P* value was calculated for each SNP from *Z*_combined_. The beta coefficient (β) for each SNP was obtained as an inverse-variance weighted estimator, and the standard error (SE) of β was as a square root of the variance as follows:

$${{\varvec{\beta}}}_{\mathbf{c}\mathbf{o}\mathbf{m}\mathbf{b}\mathbf{i}\mathbf{n}\mathbf{e}\mathbf{d}}=\frac{{{\varvec{\beta}}}_{\mathbf{J}\mathbf{a}\mathbf{p}\mathbf{a}\mathbf{n}\mathbf{e}\mathbf{s}\mathbf{e}}/{{{\varvec{S}}\mathbf{E}}_{\mathbf{J}\mathbf{a}\mathbf{p}\mathbf{a}\mathbf{n}\mathbf{e}\mathbf{s}\mathbf{e}}}^{2}+{{\varvec{\beta}}}_{\mathbf{i}\mathbf{n}\mathbf{t}\mathbf{e}\mathbf{r}\mathbf{n}\mathbf{a}\mathbf{t}\mathbf{i}\mathbf{o}\mathbf{n}\mathbf{a}\mathbf{l}}/{{\mathbf{S}\mathbf{E}}_{\mathbf{i}\mathbf{n}\mathbf{t}\mathbf{e}\mathbf{r}\mathbf{n}\mathbf{a}\mathbf{t}\mathbf{i}\mathbf{o}\mathbf{n}\mathbf{a}\mathbf{l}}}^{2}}{1/{{\mathbf{S}\mathbf{E}}_{\mathbf{J}\mathbf{a}\mathbf{p}\mathbf{a}\mathbf{n}\mathbf{e}\mathbf{s}\mathbf{e}}}^{2}+1/{{\mathbf{S}\mathbf{E}}_{\mathbf{i}\mathbf{n}\mathbf{t}\mathbf{e}\mathbf{r}\mathbf{n}\mathbf{a}\mathbf{t}\mathbf{i}\mathbf{o}\mathbf{n}\mathbf{a}\mathbf{l}}}^{2}}$$

and

$${\mathbf{S}\mathbf{E}}_{\mathbf{c}\mathbf{o}\mathbf{m}\mathbf{b}\mathbf{i}\mathbf{n}\mathbf{e}\mathbf{d}}=\sqrt{\frac{1}{1/{{\mathbf{S}\mathbf{E}}_{\mathbf{J}\mathbf{a}\mathbf{p}\mathbf{a}\mathbf{n}\mathbf{e}\mathbf{s}\mathbf{e}}}^{2}+1/{{\mathbf{S}\mathbf{E}}_{\mathbf{i}\mathbf{n}\mathbf{t}\mathbf{e}\mathbf{r}\mathbf{n}\mathbf{a}\mathbf{t}\mathbf{i}\mathbf{o}\mathbf{n}\mathbf{a}\mathbf{l}}}^{2}}}\boldsymbol{ }\boldsymbol{ }.$$

To explore known functional effects of lead SNPs and candidate SNPs, FUMA-GWAS v1.3.7 was used^26^. The 1000G phase3 SAS was selected as the reference panel population, and the lead and candidate SNPs were determined with the default values for all other parameters.

### Web resources

GWAS data in this study will be submitted to the NBDC Human Database, a public database in Japan (NBDC Human Database, https://humandbs.biosciencedbc.jp/en/) and the Japanese Genotype–phenotype Archive (JGA).

## Supplementary Information

Supplementary Information.
