# Supplementary material for: Genetic association of IL17 and the importance of ABO blood group antigens in saliva to COVID-19
Source: Sci Rep. 2022 Mar 9;12:3854. doi: 10.1038/s41598-022-07856-3 (PMC8907215; doi:10.1038/s41598-022-07856-3)
Supplement: Supplementary file 1 — Supplementary Information. [file 41598_2022_7856_MOESM1_ESM.docx]

Supplementary Material

**Genetic association of IL17 and the importance of ABO blood group antigens in saliva to COVID-19**

**Nao Nishida, Masaya Sugiyama, Yosuke Kawai, Izumi Naka, Noriko Iwamoto, Tetsuya Suzuki, Michiyo Suzuki, Yusuke Miyazato, Satoshi Suzuki, Shinyu Izumi, Masayuki Hojo, Takayo Tsuchiura, Miyuki Ishikawa, Jun Ohashi, Norio Ohmagari, Katsushi Tokunaga, Masashi Mizokami**

**Supplementary Table 1.** **Significantly associated markers in the integrated GWAS comparing all COVID-19 patients and the general population.**

| rsid | Nearest  gene | chr | position  (hg19) | A1 | Japanese GWAS | | | International meta GWAS | | | Integrated GWAS | | |
| --- | --- | --- | --- | --- | --- | --- | --- | --- | --- | --- | --- | --- | --- |
|  |  |  |  |  | OR | 95%CI | P | meta_OR | 95%CI | meta_P | meta_OR | 95%CI | meta_P |
| rs2894439 | FOXP4-AS1 | 6 | 41480093 | A | 1.32 | 1.13-1.55 | 4.54E-04 | 1.21 | 1.11-1.33 | 1.70E-05 | 1.24 | 1.15-1.34 | 3.38E-08 |
| rs9367106 | FOXP4-AS1 | 6 | 41483390 | C | 1.34 | 1.15-1.57 | 2.82E-04 | 1.21 | 1.11-1.33 | 2.16E-05 | 1.24 | 1.15-1.34 | 2.53E-08 |
| rs12660421 | FOXP4-AS1 | 6 | 41488378 | A | 1.37 | 1.17-1.61 | 1.10E-04 | 1.16 | 1.08-1.24 | 5.55E-05 | 1.19 | 1.12-1.27 | 2.34E-08 |
| rs1853837 | FOXP4-AS1 | 6 | 41497035 | A | 1.40 | 1.19-1.65 | 6.59E-05 | 1.15 | 1.08-1.21 | 1.94E-06 | 1.17 | 1.11-1.23 | 6.12E-10 |
| rs55889968 | FOXP4-AS1 | 6 | 41501225 | G | 1.44 | 1.22-1.7 | 1.25E-05 | 1.17 | 1.08-1.27 | 7.92E-05 | 1.22 | 1.14-1.31 | 4.10E-09 |
| rs12175265 | FOXP4-AS1 | 6 | 41501834 | A | 1.44 | 1.22-1.7 | 1.25E-05 | 1.17 | 1.09-1.26 | 2.29E-05 | 1.21 | 1.13-1.29 | 1.18E-09 |
| rs1886814 | FOXP4-AS1 | 6 | 41502683 | C | 1.43 | 1.22-1.69 | 1.82E-05 | 1.25 | 1.15-1.35 | 3.44E-08 | 1.28 | 1.19-1.38 | 4.15E-12 |
| rs4714474 | FOXP4-AS1 | 6 | 41503561 | A | 1.44 | 1.22-1.7 | 1.25E-05 | 1.17 | 1.09-1.26 | 4.85E-05 | 1.22 | 1.13-1.3 | 2.49E-09 |
| rs9381074 | FOXP4-AS1 | 6 | 41505196 | A | 1.44 | 1.22-1.69 | 1.63E-05 | 1.24 | 1.15-1.34 | 5.41E-08 | 1.28 | 1.19-1.37 | 5.48E-12 |
| rs8176719 | ABO | 9 | 136132908 | TC | 1.26 | 1.08-1.47 | 2.95E-03 | 1.10 | 1.07-1.14 | 3.66E-08 | 1.11 | 1.07-1.15 | 2.02E-09 |
| rs2248420 | IFNAR2 | 21 | 34605778 | C | 0.88 | 0.75-1.03 | 9.87E-02 | 0.88 | 0.85-0.91 | 3.77E-14 | 0.88 | 0.85-0.91 | 7.06E-11 |
| rs17860142 | IFNAR2 | 21 | 34607870 | C | 0.88 | 0.75-1.03 | 9.87E-02 | 0.88 | 0.85-0.91 | 2.92E-14 | 0.88 | 0.85-0.91 | 6.04E-11 |
| rs3153 | IFNAR2 | 21 | 34609505 | G | 0.88 | 0.75-1.03 | 9.87E-02 | 0.88 | 0.85-0.91 | 1.58E-13 | 0.88 | 0.85-0.91 | 1.70E-10 |
| rs12482014 | IFNAR2 | 21 | 34611318 | C | 0.88 | 0.75-1.03 | 9.87E-02 | 0.88 | 0.85-0.91 | 1.08E-13 | 0.88 | 0.85-0.91 | 1.35E-10 |
| rs12482193 | IFNAR2 | 21 | 34611545 | T | 0.88 | 0.75-1.03 | 9.87E-02 | 0.88 | 0.85-0.91 | 3.26E-14 | 0.88 | 0.85-0.91 | 6.46E-11 |
| rs12482060 | IFNAR2 | 21 | 34611571 | C | 0.88 | 0.75-1.02 | 9.69E-02 | 0.87 | 0.84-0.9 | 1.10E-16 | 0.87 | 0.84-0.9 | 1.95E-12 |
| rs17860165 | IFNAR2 | 21 | 34611730 | C | 0.88 | 0.75-1.03 | 9.87E-02 | 0.88 | 0.85-0.91 | 8.71E-14 | 0.88 | 0.85-0.91 | 1.18E-10 |

**Supplementary Table 2. Significantly associated markers in the integrated GWAS comparing sCOVID-19 patients and the general population.**

| rsid | nearest gene | chr | position (hg19) | A1 | Japanese GWAS | | | International meta GWAS | | | Integrated GWAS | | |
| --- | --- | --- | --- | --- | --- | --- | --- | --- | --- | --- | --- | --- | --- |
|  |  |  |  |  | OR | 95%CI | P | meta_OR | 95%CI | meta_P | meta_OR | 95%CI | meta_P |
| rs1853837 | FOXP4-AS1 | 6 | 41497035 | A | 1.84 | 1.37-2.47 | 5.41E-05 | 1.24 | 1.13-1.37 | 1.52E-05 | 1.29 | 1.18-1.42 | 3.35E-09 |
| rs2186317 | IFNAR2 | 21 | 34593574 | G | 0.71 | 0.51-0.98 | 3.84E-02 | 0.87 | 0.82-0.91 | 6.90E-09 | 0.86 | 0.82-0.9 | 2.69E-08 |
| rs62226132 | IFNAR2 | 21 | 34593710 | T | 0.74 | 0.55-1.01 | 5.89E-02 | 0.85 | 0.8-0.89 | 4.18E-10 | 0.84 | 0.8-0.89 | 8.77E-09 |
| rs62226152 | IFNAR2 | 21 | 34596750 | C | 0.74 | 0.55-1.01 | 5.89E-02 | 0.85 | 0.8-0.89 | 4.44E-10 | 0.84 | 0.8-0.89 | 9.12E-09 |
| rs4553897 | IFNAR2 | 21 | 34599084 | G | 0.74 | 0.55-1.01 | 5.89E-02 | 0.84 | 0.8-0.89 | 2.65E-10 | 0.84 | 0.8-0.89 | 6.51E-09 |
| rs12482556 | IFNAR2 | 21 | 34602934 | T | 0.73 | 0.54-0.99 | 4.29E-02 | 0.84 | 0.8-0.88 | 1.64E-11 | 0.84 | 0.79-0.88 | 5.86E-10 |
| rs11088247 | IFNAR2 | 21 | 34603249 | C | 0.73 | 0.54-0.99 | 4.29E-02 | 0.84 | 0.8-0.88 | 3.30E-11 | 0.84 | 0.8-0.88 | 9.25E-10 |
| rs2300370 | IFNAR2 | 21 | 34604557 | G | 0.73 | 0.54-0.99 | 4.29E-02 | 0.84 | 0.79-0.88 | 6.90E-12 | 0.83 | 0.79-0.88 | 3.33E-10 |
| rs2248420 | IFNAR2 | 21 | 34605778 | C | 0.73 | 0.54-0.99 | 4.22E-02 | 0.84 | 0.8-0.89 | 9.45E-11 | 0.84 | 0.8-0.88 | 1.80E-09 |
| rs2834154 | IFNAR2 | 21 | 34606634 | A | 0.73 | 0.54-0.99 | 4.29E-02 | 0.84 | 0.8-0.89 | 5.57E-11 | 0.84 | 0.8-0.88 | 1.30E-09 |
| rs6517153 | IFNAR2 | 21 | 34607436 | G | 0.73 | 0.54-0.99 | 4.29E-02 | 0.84 | 0.8-0.89 | 4.07E-11 | 0.84 | 0.8-0.88 | 1.06E-09 |
| rs17860142 | IFNAR2 | 21 | 34607870 | C | 0.73 | 0.54-0.99 | 4.22E-02 | 0.84 | 0.8-0.89 | 9.44E-11 | 0.84 | 0.8-0.88 | 1.79E-09 |
| rs3153 | IFNAR2 | 21 | 34609505 | G | 0.73 | 0.54-0.99 | 4.22E-02 | 0.84 | 0.8-0.89 | 2.76E-10 | 0.84 | 0.8-0.89 | 3.65E-09 |
| rs9636867 | IFNAR2 | 21 | 34609944 | A | 0.73 | 0.54-0.99 | 4.29E-02 | 0.84 | 0.79-0.88 | 7.57E-12 | 0.83 | 0.79-0.88 | 3.54E-10 |
| rs1131964 | IFNAR2 | 21 | 34610487 | T | 0.73 | 0.53-1 | 4.68E-02 | 0.86 | 0.82-0.9 | 3.80E-10 | 0.85 | 0.81-0.89 | 5.42E-09 |
| rs12482014 | IFNAR2 | 21 | 34611318 | C | 0.73 | 0.54-0.99 | 4.22E-02 | 0.84 | 0.8-0.89 | 1.56E-10 | 0.84 | 0.8-0.88 | 2.50E-09 |
| rs12482193 | IFNAR2 | 21 | 34611545 | T | 0.73 | 0.54-0.99 | 4.22E-02 | 0.84 | 0.8-0.88 | 5.43E-11 | 0.84 | 0.79-0.88 | 1.25E-09 |
| rs12482060 | IFNAR2 | 21 | 34611571 | C | 0.73 | 0.54-0.99 | 4.04E-02 | 0.84 | 0.8-0.89 | 9.94E-11 | 0.84 | 0.8-0.88 | 1.71E-09 |
| rs17860165 | IFNAR2 | 21 | 34611730 | C | 0.73 | 0.54-0.99 | 4.22E-02 | 0.84 | 0.8-0.89 | 1.00E-10 | 0.84 | 0.8-0.88 | 1.86E-09 |
| rs17860169 | IFNAR2 | 21 | 34613301 | A | 0.73 | 0.54-0.99 | 4.29E-02 | 0.84 | 0.8-0.88 | 1.08E-11 | 0.83 | 0.79-0.88 | 4.46E-10 |
| rs1051393 | IFNAR2 | 21 | 34614255 | T | 0.74 | 0.55-1.01 | 5.36E-02 | 0.85 | 0.8-0.89 | 1.69E-10 | 0.84 | 0.8-0.89 | 4.07E-09 |
| rs9976829 | IFNAR2 | 21 | 34614834 | A | 0.73 | 0.54-0.99 | 4.29E-02 | 0.82 | 0.78-0.86 | 2.05E-14 | 0.82 | 0.78-0.86 | 7.95E-12 |
| rs2834157 | IFNAR2 | 21 | 34616545 | G | 0.74 | 0.55-1 | 5.28E-02 | 0.82 | 0.78-0.87 | 3.34E-13 | 0.82 | 0.78-0.86 | 7.17E-11 |
| rs2236756 | IFNAR2 | 21 | 34616923 | A | 0.73 | 0.54-0.99 | 4.29E-02 | 0.82 | 0.78-0.87 | 1.26E-13 | 0.82 | 0.78-0.86 | 2.53E-11 |
| rs2252639 | IFNAR2 | 21 | 34617729 | G | 0.74 | 0.55-1.01 | 5.36E-02 | 0.82 | 0.78-0.87 | 8.80E-14 | 0.82 | 0.78-0.86 | 3.18E-11 |
| rs2252650 | IFNAR2 | 21 | 34617950 | T | 0.74 | 0.55-1.01 | 5.36E-02 | 0.82 | 0.78-0.87 | 1.05E-13 | 0.82 | 0.78-0.86 | 3.55E-11 |
| rs2284549 | IFNAR2 | 21 | 34618043 | T | 0.74 | 0.55-1 | 5.28E-02 | 0.82 | 0.78-0.87 | 2.23E-13 | 0.82 | 0.78-0.86 | 5.55E-11 |
| rs2284550 | IFNAR2 | 21 | 34618285 | A | 0.74 | 0.55-1.01 | 5.36E-02 | 0.85 | 0.81-0.89 | 2.60E-11 | 0.85 | 0.81-0.89 | 1.21E-09 |
| rs2284551 | IFNAR2 | 21 | 34618313 | G | 0.74 | 0.55-1 | 5.28E-02 | 0.82 | 0.78-0.87 | 2.33E-13 | 0.82 | 0.78-0.86 | 5.70E-11 |
| rs12053666 | IFNAR2 | 21 | 34618439 | G | 0.74 | 0.55-1 | 5.28E-02 | 0.84 | 0.8-0.89 | 9.92E-11 | 0.84 | 0.8-0.88 | 2.80E-09 |
| rs2073361 | IFNAR2 | 21 | 34619445 | G | 0.74 | 0.55-1.01 | 5.36E-02 | 0.82 | 0.78-0.86 | 5.27E-14 | 0.82 | 0.78-0.86 | 2.30E-11 |
| rs2834161 | IFNAR2 | 21 | 34620207 | T | 0.74 | 0.55-1.01 | 5.36E-02 | 0.82 | 0.78-0.87 | 9.31E-14 | 0.82 | 0.78-0.86 | 3.29E-11 |
| rs2834163 | IFNAR2 | 21 | 34620451 | A | 0.74 | 0.55-1.01 | 5.36E-02 | 0.82 | 0.78-0.86 | 5.28E-14 | 0.82 | 0.78-0.86 | 2.30E-11 |
| rs2834164 | IFNAR2 | 21 | 34621948 | C | 0.74 | 0.55-1.01 | 5.36E-02 | 0.85 | 0.81-0.89 | 2.24E-11 | 0.85 | 0.81-0.89 | 1.09E-09 |
| rs2834165 | IFNAR2 | 21 | 34622536 | G | 0.73 | 0.54-0.99 | 4.29E-02 | 0.85 | 0.81-0.89 | 2.25E-11 | 0.85 | 0.81-0.89 | 7.20E-10 |
| rs2236757 | IFNAR2 | 21 | 34624917 | G | 0.74 | 0.55-1 | 5.28E-02 | 0.82 | 0.78-0.87 | 2.45E-13 | 0.82 | 0.78-0.86 | 5.89E-11 |

**Supplementary Table 3. Significantly associated markers in the integrated GWAS comparing sCOVID-19 and mCOVID-19 patients.**

| rsid | nearest gene | chr | position (hg19) | A1 | Japanese GWAS | | | International meta GWAS | | | Integrated GWAS | | |
| --- | --- | --- | --- | --- | --- | --- | --- | --- | --- | --- | --- | --- | --- |
|  |  |  |  |  | OR | 95%CI | P | meta_OR | 95%CI | meta_P | meta_OR | 95%CI | meta_P |
| rs11962054 | IL17A | 6 | 52061133 | A | 2.46 | 1.7-3.56 | 2.00E-06 | 2.33 | 1.37-3.96 | 1.72E-03 | 2.42 | 1.78-3.27 | 2.44E-08 |
| rs13192563 | IL17A | 6 | 52065236 | G | 2.46 | 1.7-3.56 | 2.00E-06 | 1.91 | 1.29-2.81 | 1.09E-03 | 2.18 | 1.67-2.85 | 1.42E-08 |
| rs13192246 | IL17A | 6 | 52065246 | A | 2.46 | 1.7-3.56 | 2.00E-06 | 1.91 | 1.29-2.81 | 1.09E-03 | 2.18 | 1.67-2.85 | 1.42E-08 |
| rs9474169 | IL17A | 6 | 52069137 | T | 2.46 | 1.7-3.56 | 2.00E-06 | 1.87 | 1.27-2.77 | 1.64E-03 | 2.16 | 1.65-2.83 | 2.30E-08 |

**Supplementary Table 4. SNP functions for significantly associated markers in the integrated GWAS.**

|  |  |  |  |  |  | SNP annotations | | | |
| --- | --- | --- | --- | --- | --- | --- | --- | --- | --- |
| Comparison | rsid | nearest gene | #chr | position (hg19) | A1 | Lead/Ind. Sig. SNPs | Annotation | CADD | RDB |
| i) | rs2894439 | FOXP4-AS1 | 6 | 41480093 | A | Lead SNP | ncRNA_intronic | 0.776 | 4 |
| i) | rs9367106 | FOXP4-AS1 | 6 | 41483390 | C | Independent significant SNPs | ncRNA_intronic | 6.663 | 6 |
| i) | rs12660421 | FOXP4-AS1 | 6 | 41488378 | A | Independent significant SNPs | ncRNA_intronic | 0.608 | 7 |
| i) | rs1853837 | FOXP4-AS1 | 6 | 41497035 | A | Independent significant SNPs | ncRNA_intronic | 0.54 | 5 |
| i) | rs55889968 | FOXP4-AS1 | 6 | 41501225 | G | Independent significant SNPs | ncRNA_intronic | 0.278 | 3a |
| i) | rs12175265 | FOXP4-AS1 | 6 | 41501834 | A | Independent significant SNPs | ncRNA_intronic | 3.039 | 7 |
| i) | rs1886814 | FOXP4-AS1 | 6 | 41502683 | C | Lead SNP | ncRNA_intronic | 4.015 | 5 |
| i) | rs4714474 | FOXP4-AS1 | 6 | 41503561 | A | Independent significant SNPs | ncRNA_intronic | 0.125 | 4 |
| i) | rs9381074 | FOXP4-AS1 | 6 | 41505196 | A | Independent significant SNPs | ncRNA_intronic | 4.556 | 4 |
| i) | rs8176719 | ABO | 9 | 136132908 | TC | Lead SNP | ncRNA_exonic | 15.84 | NA |
| i) | rs2248420 | IFNAR2 | 21 | 34605778 | C | Independent significant SNPs | intronic | 3.557 | 5 |
| i) | rs17860142 | IFNAR2 | 21 | 34607870 | C | Independent significant SNPs | intronic | 0.12 | 7 |
| i) | rs3153 | IFNAR2 | 21 | 34609505 | G | Independent significant SNPs | intronic | 7.596 | 5 |
| i) | rs12482014 | IFNAR2 | 21 | 34611318 | C | Independent significant SNPs | intronic | 0.603 | 4 |
| i) | rs12482193 | IFNAR2 | 21 | 34611545 | T | Independent significant SNPs | intronic | 9.456 | 1f |
| i) | rs12482060 | IFNAR2 | 21 | 34611571 | C | Lead SNP | intronic | 10.94 | 3a |
| i) | rs17860165 | IFNAR2 | 21 | 34611730 | C | Independent significant SNPs | intronic | 2.365 | 3a |
| ii) | rs1853837 | FOXP4-AS1 | 6 | 41497035 | A | Lead SNP | ncRNA_intronic | 0.54 | 5 |
| ii) | rs2186317 | IFNAR2 | 21 | 34593574 | G | Independent significant SNPs | intergenic | 0.547 | 6 |
| ii) | rs62226132 | IFNAR2 | 21 | 34593710 | T | Independent significant SNPs | intergenic | 6.1 | 6 |
| ii) | rs62226152 | IFNAR2 | 21 | 34596750 | C | Independent significant SNPs | intergenic | 7.379 | 7 |
| ii) | rs4553897 | IFNAR2 | 21 | 34599084 | G | Independent significant SNPs | intergenic | 1.459 | 7 |
| ii) | rs12482556 | IFNAR2 | 21 | 34602934 | T | Independent significant SNPs | intronic | 8.933 | 4 |
| ii) | rs11088247 | IFNAR2 | 21 | 34603249 | C | Independent significant SNPs | intronic | 6.718 | 1f |
| ii) | rs2300370 | IFNAR2 | 21 | 34604557 | G | Independent significant SNPs | intronic | 0.513 | 5 |
| ii) | rs2248420 | IFNAR2 | 21 | 34605778 | C | Independent significant SNPs | intronic | 3.557 | 5 |
| ii) | rs2834154 | IFNAR2 | 21 | 34606634 | A | Independent significant SNPs | intronic | 6.533 | 7 |
| ii) | rs6517153 | IFNAR2 | 21 | 34607436 | G | Independent significant SNPs | intronic | 3.125 | 2b |
| ii) | rs17860142 | IFNAR2 | 21 | 34607870 | C | Independent significant SNPs | intronic | 0.12 | 7 |
| ii) | rs3153 | IFNAR2 | 21 | 34609505 | G | Independent significant SNPs | intronic | 7.596 | 5 |
| ii) | rs9636867 | IFNAR2 | 21 | 34609944 | A | Independent significant SNPs | intronic | 0.257 | 3a |
| ii) | rs1131964 | IFNAR2 | 21 | 34610487 | T | Lead SNP | intronic | 3.253 | 1f |
| ii) | rs12482014 | IFNAR2 | 21 | 34611318 | C | Independent significant SNPs | intronic | 0.603 | 4 |
| ii) | rs12482193 | IFNAR2 | 21 | 34611545 | T | Independent significant SNPs | intronic | 9.456 | 1f |
| ii) | rs12482060 | IFNAR2 | 21 | 34611571 | C | Independent significant SNPs | intronic | 10.94 | 3a |
| ii) | rs17860165 | IFNAR2 | 21 | 34611730 | C | Independent significant SNPs | intronic | 2.365 | 3a |
| ii) | rs17860169 | IFNAR2 | 21 | 34613301 | A | Independent significant SNPs | intronic | 0.239 | 6 |
| ii) | rs1051393 | IFNAR2 | 21 | 34614255 | T | Independent significant SNPs | exonic | 0.885 | NA |
| ii) | rs9976829 | IFNAR2 | 21 | 34614834 | A | Lead SNP | intronic | 0.322 | 7 |
| ii) | rs2834157 | IFNAR2 | 21 | 34616545 | G | Independent significant SNPs | intronic | 6.034 | 4 |
| ii) | rs2236756 | IFNAR2 | 21 | 34616923 | A | Independent significant SNPs | intronic | 3.648 | 4 |
| ii) | rs2252639 | IFNAR2 | 21 | 34617729 | G | Independent significant SNPs | intronic | 4.568 | 4 |
| ii) | rs2252650 | IFNAR2 | 21 | 34617950 | T | Independent significant SNPs | intronic | 0.686 | 1f |
| ii) | rs2284549 | IFNAR2 | 21 | 34618043 | T | Independent significant SNPs | intronic | 4.582 | 3a |
| ii) | rs2284550 | IFNAR2 | 21 | 34618285 | A | Independent significant SNPs | intronic | 11.02 | 1f |
| ii) | rs2284551 | IFNAR2 | 21 | 34618313 | G | Independent significant SNPs | intronic | 3.45 | 5 |
| ii) | rs12053666 | IFNAR2 | 21 | 34618439 | G | Independent significant SNPs | intronic | 0.102 | 7 |
| ii) | rs2073361 | IFNAR2 | 21 | 34619445 | G | Independent significant SNPs | intronic | 7.734 | 5 |
| ii) | rs2834161 | IFNAR2 | 21 | 34620207 | T | Independent significant SNPs | intronic | 0.066 | 4 |
| ii) | rs2834163 | IFNAR2 | 21 | 34620451 | A | Independent significant SNPs | intronic | 5.091 | 6 |
| ii) | rs2834164 | IFNAR2 | 21 | 34621948 | C | Independent significant SNPs | intronic | 0.81 | 1f |
| ii) | rs2834165 | IFNAR2 | 21 | 34622536 | G | Independent significant SNPs | intronic | 0.42 | 6 |
| ii) | rs2236757 | IFNAR2 | 21 | 34624917 | G | Independent significant SNPs | intronic | 7.158 | 6 |
| iii) | rs11962054 | IL17A | 6 | 52061133 | A | Independent significant SNP | intergenic | 12.46 | 6 |
| iii) | rs13192563 | IL17A | 6 | 52065236 | G | Independent significant SNP | intergenic | 0.701 | 5 |
| iii) | rs13192246 | IL17A | 6 | 52065246 | A | LEAD SNP | intergenic | 6.412 | 5 |
| iii) | rs9474169 | IL17A | 6 | 52069137 | T | Independent significant SNP | intergenic | 1.323 | 6 |

CADD: CADD score, RDB: RegulomeDB score

**Supplementary Table 5. Association of FUT2 genotypes with development of COVID-19.**

| FUT2 rs1047781 genotype,  secretor status |  | COVID | |  | HC | |  |  |  |  |  |  |
| --- | --- | --- | --- | --- | --- | --- | --- | --- | --- | --- | --- | --- |
|  |  | (n=461) | |  | (n=1,193) | |  | P |  | OR | (95% CI) | |
|  |  | count | % |  | count | % |  |  |  |  | Lower | Upper |
| A/A, secretor |  | 190 | 41.2 |  | 449 | 37.6 |  | 1.80E-01 |  | 1.16 | 0.93 | 1.45 |
| A/T, secretor |  | 209 | 45.3 |  | 558 | 46.8 |  | 5.99E-01 |  | 0.94 | 0.76 | 1.17 |
| T/T, non secretor |  | 62 | 13.4 |  | 186 | 15.6 |  | 2.74E-01 |  | 0.84 | 0.62 | 1.15 |

**Supplementary Table 6. Six genetic regions that were not replicated in the Japanese GWAS comparing all COVID-19 patients with the general population.**

|  | International meta-GWAS | | | | | | | Japanese GWAS | | | | | | |
| --- | --- | --- | --- | --- | --- | --- | --- | --- | --- | --- | --- | --- | --- | --- |
| Nearest  gene | rsid | #chr | Position  (hg19) | A1 | meta_OR | 95%CI | meta_P | rsid | #chr | Position  (hg19) | A1 | OR | 95%CI | P |
| LZTFL1 | rs35081325 | 3 | 45889921 | A | 1.63 | 1.54-1.73 | 1.11E-61 | chr3:45896048:ACT:A | 3 | 45896048 | A | 1.27 | 1.01-1.59 | 4.07E-02 |
| CCHCR1 | rs111837807 | 6 | 31121232 | T | 1.17 | 1.10-1.23 | 2.13E-08 | rs147296625 | 6 | 31117573 | C | 1.65 | 1.12-2.41 | 1.06E-02 |
| TMEM65 | rs72711165 | 8 | 125336564 | T | 1.35 | 1.22-1.51 | 2.64E-08 | rs72711186 | 8 | 125375181 | C | 1.29 | 0.98-1.71 | 7.04E-02 |
| OAS1 | rs10774671 | 12 | 113357193 | G | 1.11 | 1.07-1.14 | 1.51E-09 | rs12423440 | 12 | 113356309 | T | 0.68 | 0.46-1.01 | 5.27E-02 |
| KANSL1 | rs1819040 | 17 | 44219831 | T | 0.88 | 0.84-0.92 | 9.95E-10 | rs2023861 | 17 | 44177103 | T | 0.81 | 0.69-0.96 | 1.64E-02 |
| DPP9 | rs2109069 | 19 | 4719443 | G | 1.16 | 1.12-1.20 | 5.76E-17 | rs149976999 | 19 | 4706101 | T | 1.58 | 1.02-2.45 | 4.26E-02 |

SNP showing the lowest P-value among SNPs which exist on each gene was listed in table.

**Supplementary Table 7. Six genetic regions that were not replicated in the Japanese GWAS comparing sCOVID-19 patients with the general population.**

|  | International meta-GWAS | | | | | | | Japanese GWAS | | | | | | |
| --- | --- | --- | --- | --- | --- | --- | --- | --- | --- | --- | --- | --- | --- | --- |
| Nearest  gene | rsid | #chr | position (hg19) | A1 | meta_OR | 95%CI | meta_P | rsid | #chr | position (hg19) | A1 | OR | 95%CI | P |
| LZTFL1 | rs35081325 | 3 | 45889921 | A | 1.88 | 1.73-2.05 | 2.70E-49 | rs75442536 | 3 | 45950911 | A | 1.31 | 0.92-1.87 | 1.41E-01 |
| CCHCR1 | rs111837807 | 6 | 31121232 | T | 1.32 | 1.22-1.42 | 4.71E-12 | rs9263740 | 6 | 31111400 | C | 1.38 | 1.01-1.89 | 4.56E-02 |
| VSTM2A | rs71525842 | 7 | 54647894 | A | 1.21 | 1.13-1.29 | 1.42E-08 | rs17671790 | 7 | 54625957 | G | 0.56 | 0.35-0.89 | 1.41E-02 |
| OAS3 | rs2269899 | 12 | 113381956 | C | 1.21 | 1.15-1.27 | 3.31E-13 | rs76727481 | 12 | 113385180 | A | 1.69 | 1.04-2.76 | 3.41E-02 |
| TAC4 | rs77534576 | 17 | 47940666 | C | 1.49 | 1.30-1.71 | 8.60E-09 | rs61625769 | 17 | 47915959 | C | 0.53 | 0.33-0.88 | 1.30E-02 |
| DPP9 | rs2109069 | 19 | 4719443 | G | 1.28 | 1.22-1.35 | 4.17E-21 | rs150031934 | 19 | 4701134 | T | 1.82 | 1.01-3.27 | 4.53E-02 |

SNP showing the lowest P-value among SNPs which exist on each gene was listed in table.

**Supplementary Table 8. Clinical background of 462 COVID-19 patients who passed the sample quality control in GWAS.**

|  | aCOVID-19 (n=462) | removed from the analysis (n=41) |
| --- | --- | --- |
| Age, ave. (min-max) | 47.6 (20-89) | 44.6 (23-88) |
| Sex, M/F | 250/212 | 20/21 |
| High blood pressure, n (%) | 94 (20.3%) | 7 (17.1%) |
| Dyslipidemia, n (%) | 68 (14.7%) | 3 (7.3%) |
| TIIDM, n (%) | 43 (9.3%) | 2 (4.9%) |
| Bronchial asthma, n (%) | 25 (5.4%) | 3 (7.3%) |
| Hyperuricemia, n (%) | 40 (8.7%) | 2 (4.9%) |
| Obesity, n (%) | 28 (6.1%) | 3 (7.3%) |

**Supplementary Figure 1. LocusZoom plots of 400 kb around the leading SNPs for genetic regions identified in the integrated GWAS.** a. LocusZoom plots for three genetic regions identified in a GWAS comparing all COVID-19 patients and the general population. b. LocusZoom plots for two genetic regions identified in a GWAS comparing sCOVID-19 patients and the general population. c. LocusZoom plot for the IL17A and IL17F gene region identified in a GWAS comparing severe and mild COVID-19 patients.

**
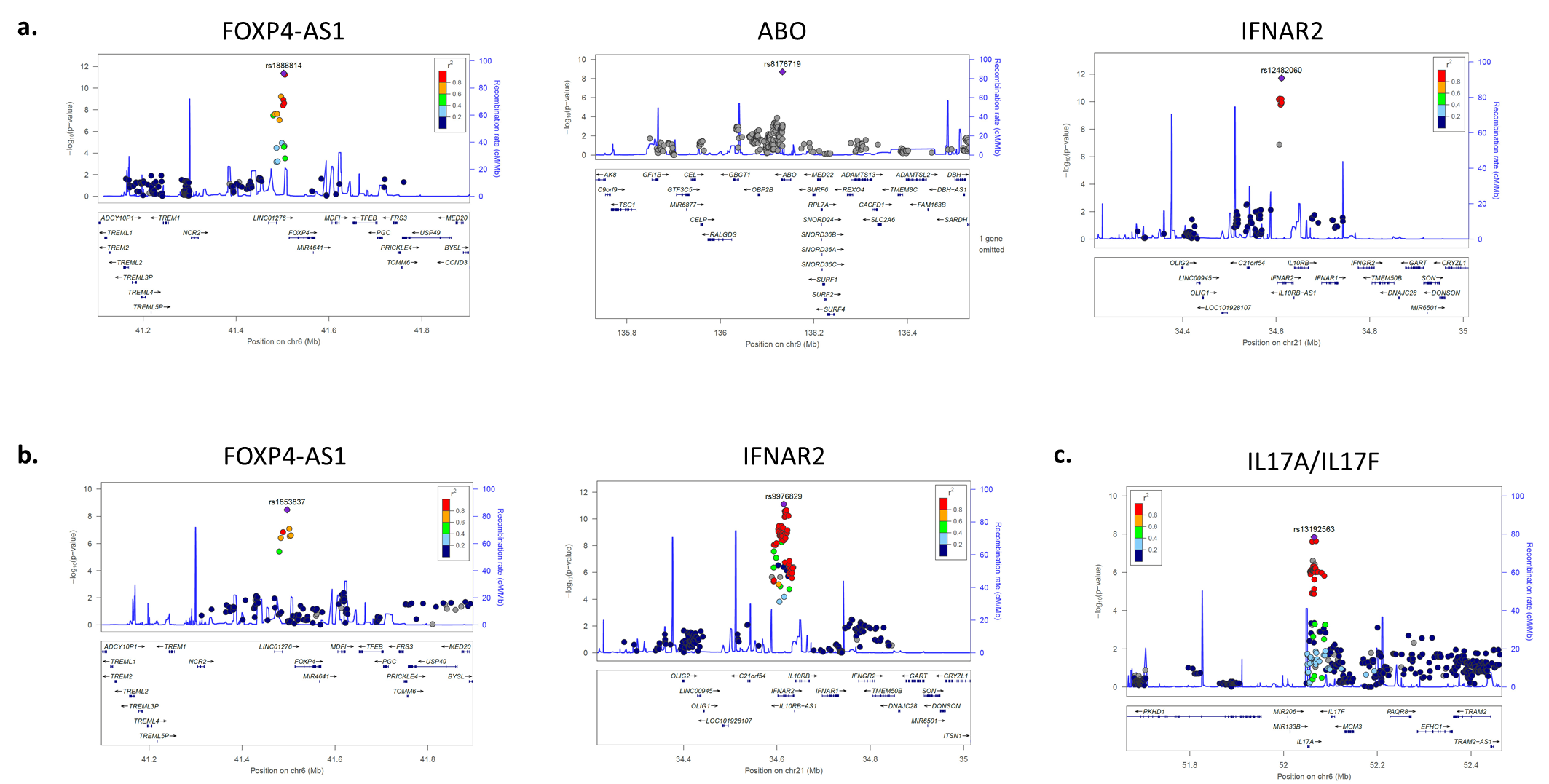
**

**Supplementary Figure 2. eQTL data of three SNPs identified in a GWAS comparing severe and mild COVID-19 patients.** The eQTL data was downloaded from the Genotype-Tissue Expression (GTEx) database.

**
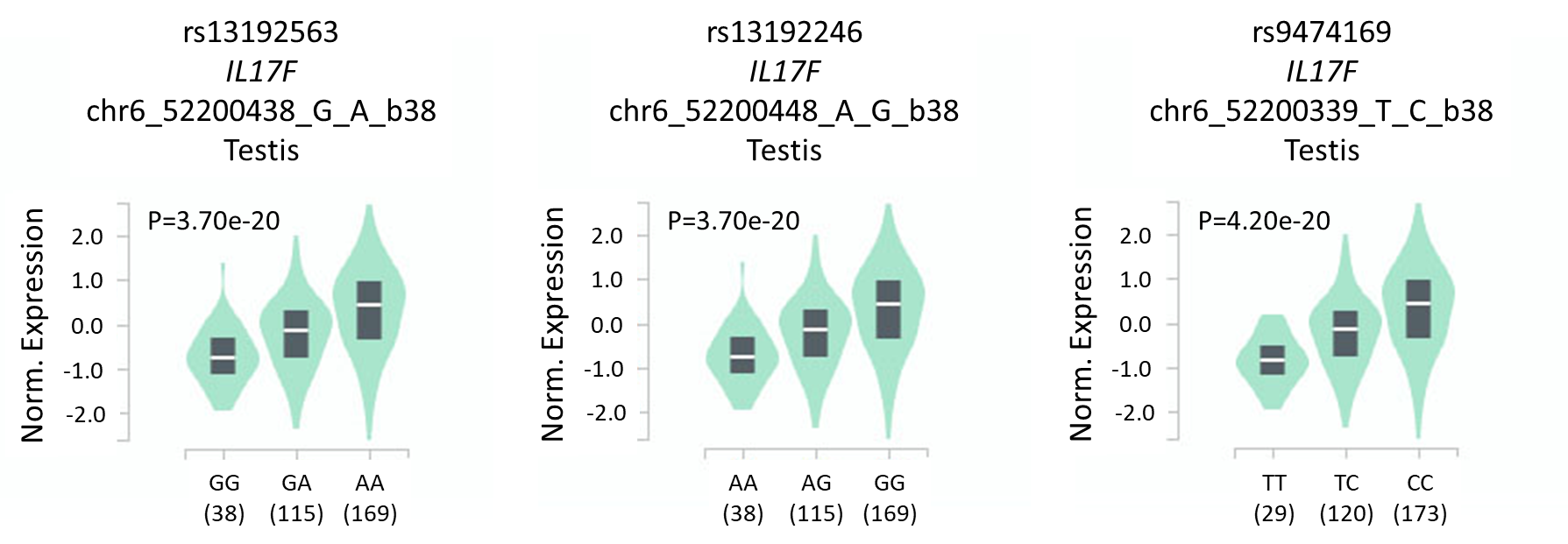
**

**Supplementary Figure 3. Principal component analysis using a total of 1,655 samples.** Genotypic principal component analysis using the first and second components was performed using 462 Japanese COVID-19 patients and 1,193 healthy individuals consisting of 400 THC and 793 PSC.

**
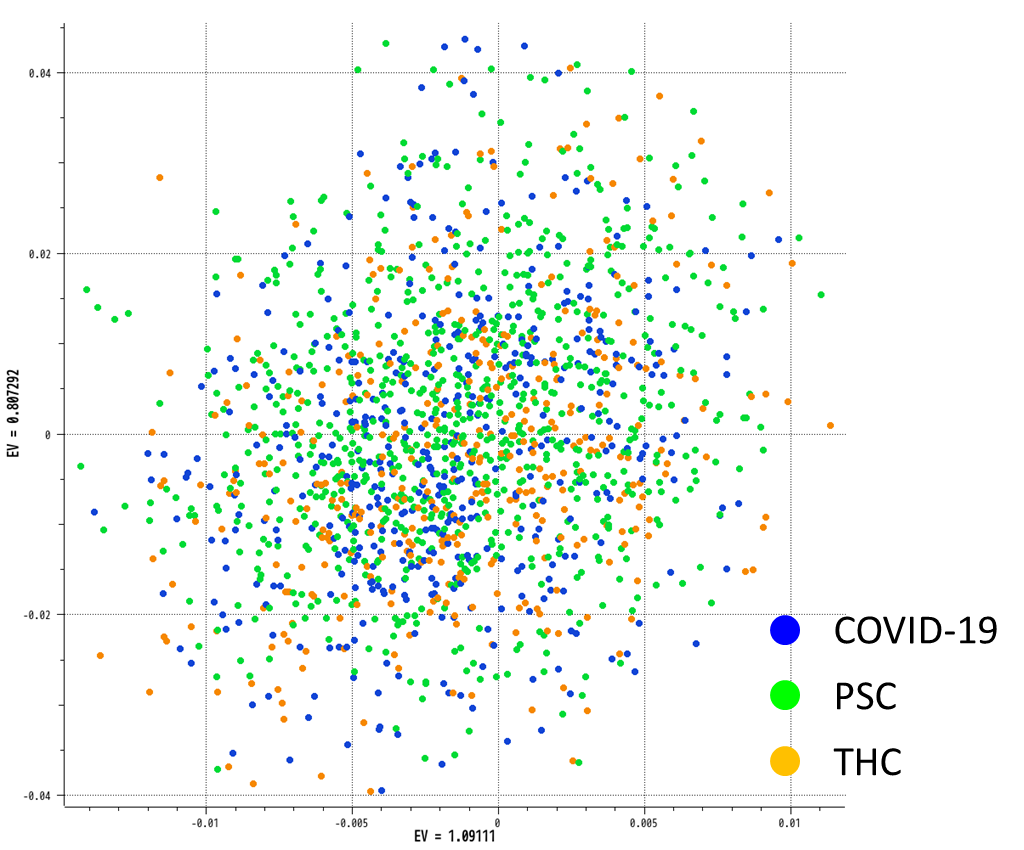
**

**Supplementary Figure 4. Principal component analysis of 1,655 samples together with 1000 Genomes.** Genotypic principal component analysis using the first and second components was performed using 1,655 samples from this study and east Asian populations (CDX, Chinese Dai in Xishuangbanna, China; CHB, Han Chinese in Beijing, China; CHS, Han Chinese South, China; JPT, Japanese in Tokyo, Japan; KHV, Kinh in Ho Chi Minh City, Vietnam) in the 1000 Genomes Project.

**
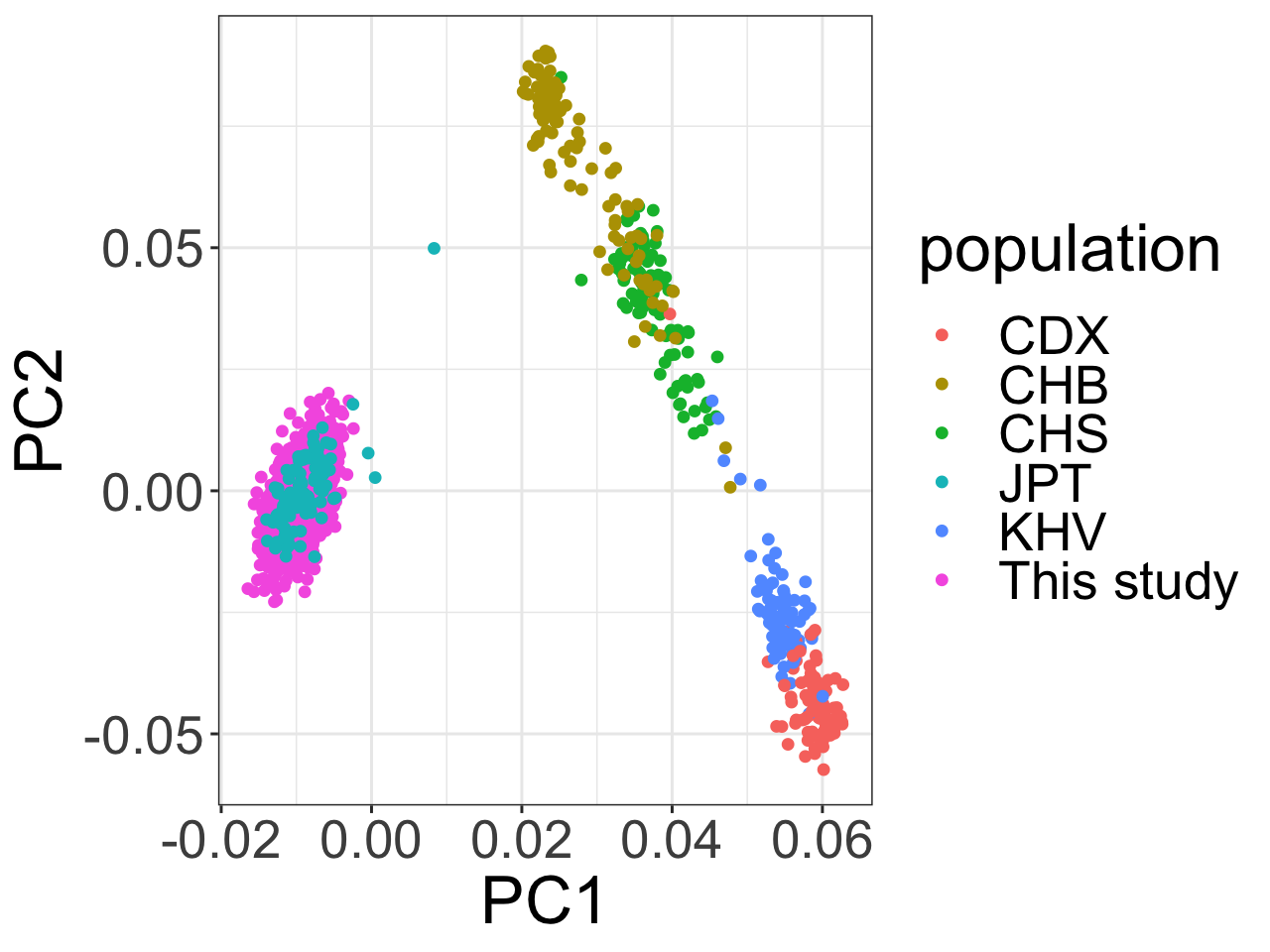
**
